# Toward resilience for public health emergency response system during COVID-19: qualitative comparative analyses of 40 countries

**DOI:** 10.3389/fpubh.2025.1652309

**Published:** 2025-09-18

**Authors:** Lingzhi Li, Yifan Tang, Mengxia Zhu, Yudi Chen, Peng Cui

**Affiliations:** ^1^Research Center of Smart City, Nanjing Tech University, Nanjing, China; ^2^Department of Management Science and Engineering, Nanjing University of Aeronautics & Astronautics, Nanjing, China; ^3^School of Civil Engineering, Nanjing Forestry University, Nanjing, China; ^4^Department of Applied Physics and Electronics, Umeå University, Umeå, Sweden

**Keywords:** COVID-19, public health emergency response system (PHERS), resilience, fuzzy set qualitative comparative analysis, configuration analysis

## Abstract

As a critical defense mechanism against COVID-19, the national public health emergency response system (PHERS) with high resilience enables effective identification, absorption, and resistance of epidemic crises. This resilience is essential for safeguarding public health and rapidly restoring social stability. However, existing studies primarily focus on single-aspect strategies in specific countries, lacking a systematic understanding of how resilience strategies influence PHERS resilience outcomes. Therefore, this study aims to establish evidence-based and configurational resilience strategies to improve the effectiveness of PHERS in responding to epidemic threats. This study proposes a theoretical framework to characterize resilience strategies and resilience outcomes for PHERS. The fuzzy-set qualitative comparative analysis (fsQCA) method is applied to analyze data from 40 countries during the COVID-19 crisis. The findings reveal three configuration paths to enhance robustness and three paths to enhance rapidity. These results emphasize the importance of the synergistic implementation of containment and closures, travel control, public personal protection, and early virus detection in improving PHERS resilience. This study provides a structured approach to understanding PHERS resilience by identifying key configuration paths that enhance robustness and rapidity. The results offer actionable insights for designing resilient PHERS to better respond to future epidemics.

## 1 Introduction

The COVID-19 pandemic has triggered an unprecedented global health crisis, resulting in extensive loss of life, economic disruption, and social instability (1). As a critical defense mechanism, Public Health Emergency Response Systems (PHERS) are collaboratively established by national governments, health organizations, and emergency rescue departments in response to public health emergencies (2). A resilient PHERS has its ability to promptly identify and absorb the detrimental effects caused by the public health crisis, while implementing effective response strategies to safeguard human life and health and swiftly restore social system stability (3). Increasing occurrences of acute public health events, such as epidemics and pandemics, highlight the importance of strengthening PHERS resilience across the world (4). However, response strategies in several countries have fallen short of expectations in enhancing PHERS resilience, failing to control the spread of the COVID-19 virus and thereby increasing the government’s financial burden. Thus, it is essential to explore effective response strategies to enhance PHERS resilience against public health shocks such as COVID-19.

Based on the widely-accepted Bruneau’s 4R theory (5), prior studies have demonstrated that the resilience outcomes of a system are determined by its robustness and rapidity capacities, whereas the means to strengthen resilience are exemplified by its redundancy and resourcefulness capacities (5). In light of this causal framework, this study characterizes the resilience outcomes of PHERS by its robustness and rapidity capacities, and explore the resilience strategies affecting these resilience outcomes in terms of redundancy and resourcefulness. Specifically, *robustness* represents the PHERS’s capacity to absorb and response to the adverse effects of COVID-19 crisis while simultaneously maintaining social stability. *Rapidity*, on the other hand, refers to the duration within which PHERS can mitigate the epidemic and restore social stability. Additionally, *redundancy* and *resourcefulness, respectively,* signify PHERS’s ability to implement alternative restriction measures—such as containment, closure, and travel control measures— and to mobilize protective and detection resources, i.e., personal protective products and virus-detection materials (6–9). Both these capacities are crucial means for controlling epidemic spread and expediting recovery during the crisis.

Prior studies have investigated the effect of various response strategies on enhancing PHERS resilience from multiple perspectives. Scholars have examined the positive effectiveness of face coverings and masks in controlling the COVID-19 pandemic across 40 countries (10). Extensive debates have also surrounded the effects of other non-pharmaceutical interventions, such as vaccination (11), travel restriction (12), school closures and work from home (13), as well as government financial support (14). These studies primarily focused on the net impact of individual policies or, at most, the interaction of two policies. However, practical evidence has clarified that effective epidemic prevention and control are driven by multiple policies rather than a single measure (15). A combination of various strategies is called configuration, which considers the interaction effects combined response strategies on PHERS resilience. Despite this, few studies focus on addressing these interaction effects and exploring the optimal configuration paths to enhance PHERS resilience. To address this gap, this study adopts fuzzy-set qualitative comparative analysis (fsQCA), a method well-suited to examining complex causality and configurations in social systems (16, 17). FsQCA allows identification of necessary and sufficient conditions and their combinations for achieving high resilience, thus providing deeper insights into the multi-strategy configurations driving PHERS resilience. The specific objectives are included as follows:

1. Select and categorize resilience strategies and resilience-outcome indicators to conceptualize their casual relationships based on the 4R theory;
2. Conduct fuzzy-set quantitative comparative analysis (fsQCA) to examine the necessary and sufficient conditions for PHERS to achieve high resilience;
3. Explore evidence-based configurational strategies for enhancing PHERS resilience and discuss their suitability for different countries.

## 2 Literature review

### 2.1 PHERS resilience concept and its capacities

The word ‘resilience’ origins from the Latin prefix ‘re-’ (back) and the verb ‘salire’ (to surge, spring). Various interpretations and aspects have been added to this term as various disciplines have adopted it gradually. The core concept of resilience emerged as the capacity of an individual, population, or system to endure a disturbance while still retaining the fundamental functions or characteristics of its initial state (18). While resilience is a fundamental concept of disaster risk reduction, its definition in the context of PHERS lacks a universally accepted standard. Most existing definitions have emphasized aspects such as preparedness and the capacity to absorb, adapt, and transform in response to acute shocks (19, 20). Despite varied interpretations, these definitions share a common core: resilience is regarded as the degree to which a system can maintain its functionality under changing circumstances (21, 22). Building on these perspectives, this study defines PHERS resilience as the “PHERS’s ability to respond to a public health emergency, including rapid response, coordination, decision-making, and adaptation, to maintain people’s health and life safety.”

In addition to elucidating the concept of PHERS resilience, many previous studies employ multiple methods to measure PHERS resilience exhaustively. For instance, the resilience to respond to risk events can be determined based on evaluation indicators in relation to the organizational, resource, and technological aspects of PHERS (23, 24). Some studies simulate real-world scenarios to comprehend PHERS’s resilience capacities, analyze the capacity gaps, identify potential risks and flaws, and establish strategies for optimizing PHERS (25, 26). Bruneau’s theory of 4R (5) (robustness, rapidity, redundancy, and resourcefulness) is extensively used in system resilience studies to represent resilience capacities (27, 28). Previous studies have demonstrated that the robustness and rapidity capacities represent the resilience of systems, while the redundancy and resourcefulness capacities depict the means to enhance resilience (29, 30). Given the causality between the 4Rs, robustness and rapidity will serve as the PHERS resilience indicators in this study. The robustness capacity emphasizes the system’s strength or its capacity to prevent propagation of damage in the presence of disruptive events (5). In this study, it refers to the capacity of PHERS to resist and respond to the COVID-19 crisis and maintain social stability, such as by limiting the number of people afflicted by the epidemic. The rapidity capacity emphasizes the rate at which a system could recover to full functionality or at least to an acceptable level of functionality following a disruption (31). The rapidity capacity in this study accentuates the ability to expeditiously recover following the PHERS to promote the social function recovery following the COVID-19 outbreak.

### 2.2 The resilience strategies of the PHERS in response to COVID-19

Due to the lack of effective COVID-19 medications, it is crucial to implement scientifically-informed policy interventions to combat the epidemic (27, 28). Consequently, an increasing number of studies concentrate on identifying effective strategies for enhancing PHERS resilience to combat COVID-19 crisis. For instance, Steffen studied the impact of COVID-19 on mask wearing by developing a zoning model to assess the community-wide impact of mask use by the asymptomatic public and concluded that masks are beneficial for both preventing disease in healthy individuals and in preventing asymptomatic transmission (32). Tim emphasizes the significance of testing during the epidemic by analyzing the role of testing during the COVID-19 pandemic (33). In addition to public personal protection and early detection strategies, the majority of existing studies also focus on unilateral strategies, such as economic strategies (34), or governance strategies (35). Nonetheless, response strategies for promoting the PHERS resilience to COVID-19 epidemic include but are not limited to personal protection requirements, an adequate supply of medical resources (31), improving the quality and capacity of medical services, and providing financial support for individuals, enterprises and affected sectors, as well as effective governance strategies (36), etc. And these strategies will interact with one another, resulting in configuration paths that enhance PHERS resilience (37, 38). Thus, it is necessary to investigate configurational resilience strategies and establish a comprehensive framework to summarize these strategies for PHERS in response to COVID-19 pandemic.

Additionally, numerous countries have published reports detailing and analyzing their responses to COVID-19 outbreak. For instance, the Chinese government has successively released dozens of versions of the Novel Coronavirus Pneumonia Prevention and Control Plan, which describes the strategies taken during the prevention and control of the epidemic, including lockdown of the city, delaying school start dates, isolating cases, and closing contacts (39). In March of 2020, the U. S. government released the U. S. Guidelines for novel coronavirus, which include providing financial and resource support, enhancing testing and diagnosis, and improving medical care (40). The European Centre for Disease Prevention and Control has established a website to offer recommendations and strategies for responding to the crisis (41). Furthermore, a number of scholars discussed single–country/region-specific responses to COVID-19. For instance, Sara et al. analyzed the response strategies in Ireland, such as universal healthcare, and examined whether and how these strategies contribute to the country’s health system reform (42). Olufadewa et al. (43) summarized the potential adaptability, efficacy, and innovative strategies from China, Italy, and the United States to assist African countries with inadequate medical systems in responding more effectively to COVID-19. However, without comparing their effectiveness in various countries, the aforementioned reports and studies directly propose the strategic policies for each national PHERS to combat the COVID-19 crisis. Without comparing multiple countries, it actually becomes difficult to determine the causal relationship between resilience strategies and resilience of PHERS, making it challenging to identify effective strategies in a scientific manner.

To bridge the aforementioned research gaps, this study selects 40 country cases and applies the fsQCA method to clarify the causal relationship between resilience strategies and resilience of PHERS by comparing the practices of different countries’ in response to the COVID-19 crisis, as well as to explore the various configuration paths of resilience strategies for PHERS to achieve high resilience for different countries. In future epidemic scenarios, this will provide researchers and decision-makers with more effective strategies for prevention and control.

## 3 Methodology

### 3.1 Study design

Qualitative comparative analysis (QCA) is a hybrid approach that seamlessly integrates qualitative and quantitative traditions (44), and it has been increasingly applied in various social science disciplines (45). Unlike conventional statistical methods, QCA is a case-oriented comparative approach, focusing on the “configurational effects” of antecedent conditions and exploring how these conditions collectively lead to an outcome from a holistic perspective (46, 47). Given the rigorous consistency assessment of set theory, fuzzy-set QCA (fsQCA) is chosen to analyze the configurational strategies for enhancing PHERS resilience. This analytical process comprises four steps: conceptual model development, data collection, necessary condition analysis, and configuration analysis.

### 3.2 Conceptual model development

**Figure 1:**
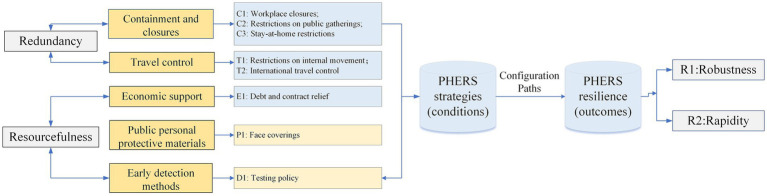
Conceptual model.

According to the 4R resilience theory (5), a theoretical model is built to conceptualize the configurational effects of resilience strategies on PHERS resilience, as illustrated in Figure 1. In this study, drawing on national experiences with the COVID-19 pandemic, eight strategies (C1–C3, T1–T2, E1, P1, and D1) are selected as condition variables, reflecting *redundancy* and *resourcefulness*. Additionally, the resilience outcomes are determined based on *robustness* and *rapidity* capacities of PHERS, quantified by the proportion of uninfected population and the average recovery time, respectively. For the calibration process in fsQCA, a direct calibration method was employed. Threshold values for full membership, the crossover point, and full non-membership were determined by combining the distributional characteristics of the dataset with relevant pandemic policy benchmarks. Specifically, the condition variables, scored on an ordinal scale (e.g., 0, 0.33, 0.67, 1), reflect the strictness level or availability of respective strategies in each country, without including a value of 0.5. The outcome variables—R1: Robustness (proportion of uninfected population) and R2: Rapidity (average recovery time)—were similarly calibrated based on empirical percentiles and theoretical resilience thresholds. The list of all 40 countries included in the study, along with the condition and outcome variables and their respective thresholds used for fsQCA, is summarized in Appendix Table S1.

### 3.3 Data collection

The data collection period for this study spans from early 2020 to late 2021, capturing the dynamic evolution and maturation of PHERS policies across multiple countries. To ensure a robust and reliable analysis of PHERS resilience over time, this research focuses on 40 countries selected based on the systematic tracking, high quality, and consistent availability of data throughout this two-year period. These countries (the names of these countries are shown in Appendix Table S1) also represent diverse geographical regions and socioeconomic backgrounds to enhance the study’s generalizability. The condition variables data were obtained from the Oxford COVID-19 Government Response Tracker (OxCGRT) database (48), while the outcome variables data came from the WHO dashboard (49), both of which are authoritative and widely used sources for COVID-19 research.

**Figure 2:**
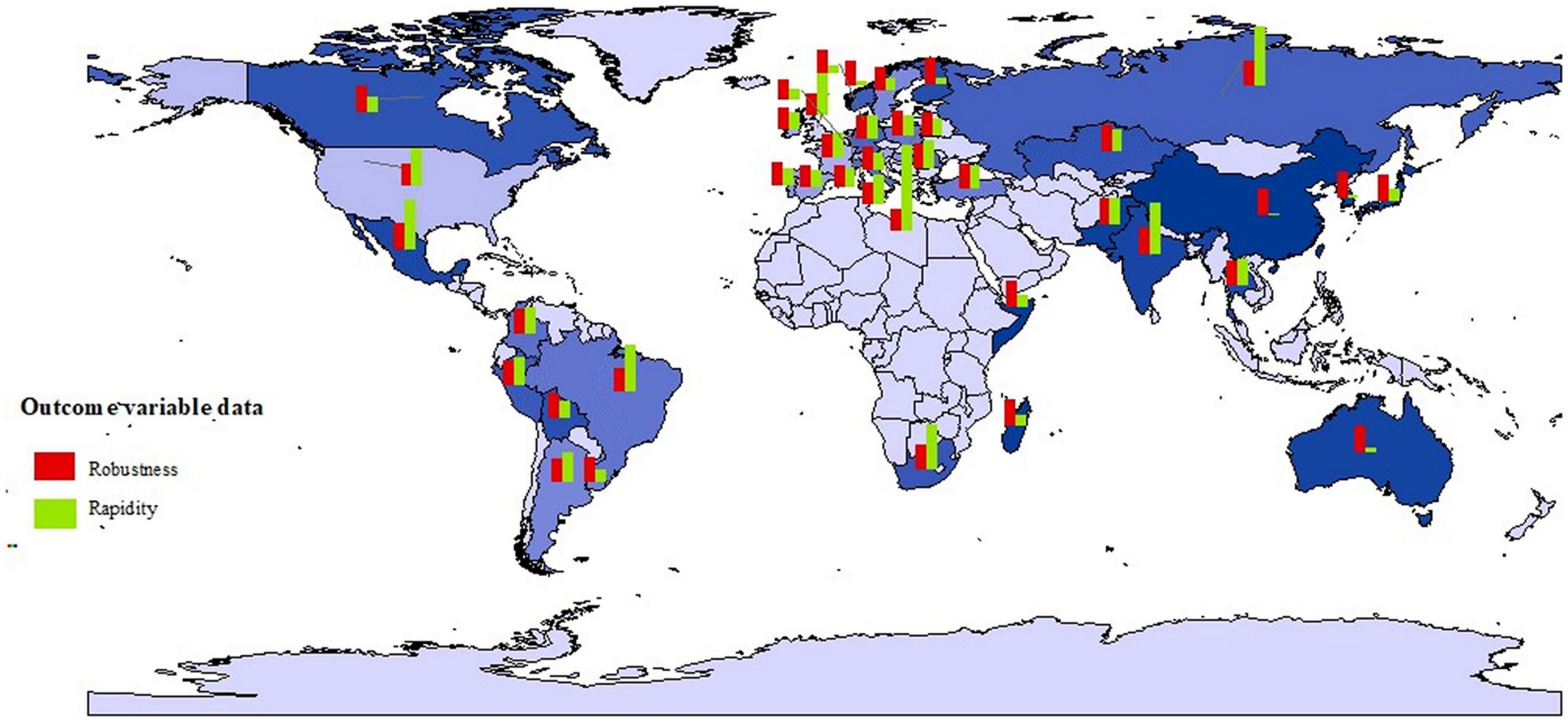
The resilience outcomes of PHERS across case countries.

The resilience outcomes of 40 countries are depicted in Figure 2. China exhibits the strongest PHERS robustness among the 40 countries, while Britain and the U. S. are less robust. China also has the shortest recovery time, making its PHERS rapidity the fastest. In contrast, Austria’s recovery took 160 days, showing a notably slow reaction compared to others.

### 3.4 Necessary condition analysis

Necessity condition analysis evaluates the explanatory power of a single condition variable on outcome variables (50). Traditionally, a condition or a combination of conditions is considered “necessary” if its consistency score exceeds 0.9 (51). In this study, necessity condition analysis evaluates the explanatory power of a single condition variable on outcome variables, specifically the robustness and rapidity of PHERS. This analysis helps to preliminarily screen for essential resilience strategies before conducting configuration analysis.

### 3.5 Configuration analysis

To identify the different combinations of resilience strategies that lead to high PHERS performance, this study conducts a configuration analysis using the fsQCA approach. Configurations sufficient for achieving two distinct outcomes, namely robustness and rapidity, are systematically extracted and labeled for clarity. Paths beginning with the prefix “R1” (such as R11, R12, R13) indicate configurations associated with high robustness, while those beginning with “R2” (such as R21, R22, R23) represent configurations that contribute to high rapidity. These labels are applied consistently throughout this study to enhance readability and support effective cross-referencing. The analysis focuses on five condition variables, which correspond to containment and closure measures, travel control, economic support, public personal protective materials, and early detection resources, and explores how these elements combine to generate resilient public health emergency response outcomes.

## 4 Results

### 4.1 Necessary conditions analysis results

In this study, no consistency value exceeds this threshold, indicating the absence of necessary conditions (Table 1). This suggests that eight strategies (condition variables) should be matched in conjunction to collectively influence PHERS resilience.

**Table 1:** Necessity analysis results of individual condition variables.

Condition variables	R1		R2	
	Consistency	Coverage	Consistency	Coverage
Workplace closures (C1)	0.500	0.434	0.585	0.508
~C1	0.500	0.590	0.415	0.489
Restrictions on public gatherings (C2)	0.734	0.489	0.734	0.489
~C2	0.267	0.533	0.267	0.533
Stay-at-home restrictions (C3)	0.367	0.688	0.317	0.594
~C3	0.634	0.432	0.684	0.466
Restrictions on internal movement (T1)	0.550	0.667	0.550	0.667
~T1	0.450	0.383	0.450	0.383
International travel control (T2)	0.550	0.492	0.585	0.523
~T2	0.450	0.510	0.416	0.471
Debt and contract relief (E1)	0.450	0.462	0.475	0.487
~E1	0.550	0.537	0.525	0.512
Face coverings (P1)	0.669	0.488	0.736	0.537
~P1	0.331	0.527	0.264	0.420
Testing policy (D1)	0.850	0.495	0.817	0.476
~D1	0.150	0.531	0.183	0.648

~ Denotes the negation of the conditions.

### 4.2 Configuration analysis results

The study conducts a sufficiency test to identify the feasible configuration paths for achieving high resilience in PHERS, as shown in Table 2. The consistency value of six configurations exceeds 0.65, indicating that these six configuration paths explain the PHERS resilience to a high degree (52). The solution consistency of 0.8299 and 0.6539 indicates that 82.99% of the cases satisfying the configuration paths R11, R12, and R13 exhibit high robustness, while 65.39% of the cases following the paths R21, R22, and R23 demonstrate high rapidity. These consistency values reflect the reliability of these configuration paths in achieving their respective outcomes (52).

**Table 2:** Configurational pathways leading to high PHERS resilience.

Causal conditions	Solutions					
	Robustness			Rapidity		
	R11	R12	R13	R21	R22	R23
Workplace closures (C1)	●	⊗	⊗	⊗		●
Restrictions on public gatherings (C2)	●	⊗	●	⊗	●	●
Stay-at-home restrictions (C3)	●	●	●		⊗	⊗
Restrictions on internal movement (T1)	●	⊗	●	⊗	⊗	⊗
International travel control (T2)		⊗	●	⊗	⊗	
Debt and contract relief (E1)	●	⊗	⊗	⊗	⊗	●
Face coverings (P1)	●	●	●	●	●	●
Testing policy (D1)	●	●	⊗	●	●	●
Consistency	0.8272	0.7519	1	0.6517	0.5732	0.5381
Raw coverage	0.158	0.05	0.05	0.1085	0.1665	0.1835
Unique coverage	0.1415	0.0335	0.05	0.0755	0.1005	0.134
Solution Coverage	0.2415			0.376		
Solution Consistency	0.8299			0.6539		

Black circles(●) indicate the presence of a condition, and circles “x” (⊗) indicate the absence of the condition and the blank space indicates “do not care” which means that the condition may or may not exist. The large circle represents the core condition, and the small circle represents the peripheral condition.

#### 4.2.1 Configuration paths for high robustness in PHERS

##### 4.2.1.1 Configuration path R11: C1*C2*C3*T1*E1*P1*D1

Configuration path R11 consists of the variables of *workplace closure (C1), restrictions on public gatherings (C2), stay-at-home restrictions (C3), internal movement restrictions (T1), debt and contract relief (E1), testing policy (P1), and face covering (D1)*. These configurational strategies underscore the need for prompt, comprehensive containment measures against COVID-19, coupled with mobility restrictions, robust virus detection, and financial support for businesses. Within this configuration, public gathering restrictions, stay-at-home restrictions, and internal movement constraints constitute the core strategies that highlight the necessity for stringent containment to prevent large-scale transmission, enhancing PHERS robustness.

Cases of China and Russia exemplify these configuration strategies. On January 23, 2020, the Chinese government issued a travel ban policy to lock down Wuhan and launched the first-level response to COVID-19 (53). Subsequently, stringent international travel restrictions were implemented to further mitigate cross-border transmission. These comprehensive strategies have markedly reduced virus spread, thereby effectively controlling the infection rate and demonstrating high robustness in PHERS. Leveraging the extensive healthcare system and PHERS legacy established during the Soviet era, Russia executed a comprehensive array of containment measures and guaranteed an ample supply of critical medical resources (54, 55). As a result, Russia has successfully controlled the infected population, demonstrating the remarkable robustness of its PHERS.

##### 4.2.1.2 Configuration path R12: ~C1* ~ C2*C3* ~ T1* ~ T2* ~ E1*P1*D1

Configuration path R12 is converted into the following variables: *~workplace closure (*~C1) * ~ restrictions on public gatherings (~C2) * stay-at-home restrictions (C3) * ~ internal movement restrictions (~T1) * ~ international travel control (~T2) * ~ debt and contract relief (~E1) * face coverings (P1) * testing policy (D1). This path reveals that even when excluding measures such as workplace closure, public-gathering restrictions, the international and domestic travel control, and the debt relief, stay-at-home restriction remain the core condition for mitigating COVID-19 transmission. Moreover, its effectiveness in ensuring PHERS robustness is significantly enhanced when combined with two supporting measures: face-covering policies and virus testing support.

Upon reviewing the specific cases, it has been determined that configuration path R12 is particularly applicable to Madagascar. Following a brief surge in cases from May to July in 2020, the incidence rate in Madagascar has been seen a substantial decline, representing the robustness of PHERS. The Madagascar government has implemented a range of control measures including localized lockdowns in high-density residential areas, mandatory isolation and virus testing for passengers, and the mandatory mask-wearing in all public spaces (56, 57).

##### 4.2.1.3 Configuration path R13: ~C1*C2*C3*T1*T2* ~ E1*P1* ~ D1

Configuration R13 is converted into the following variables: ~workplace closure (~C1) * restrictions on public gatherings (C2) * stay-at-home restrictions (C3) * internal movement restrictions (T1) * international travel control (T2) * ~ debt and contract relief (~E1) * face covering (P1) * ~ testing policy (D1). This configuration path highlights activity-restriction measures—such as limiting public gatherings, enforcing stay-at-home requirements, and restricting both domestic and international movement—as core conditions for controlling COVID-19 transmission. Additionally, the implementation of personal protective practices, particularly face covering, is deemed essential for further mitigating virus spread. Critically, this configuration path prioritizes reducing human contact while avoiding more disruptive measures like workplace closures and early virus-testing polices, offering a cost-effective strategy that balances infection mitigation with faster socioeconomic recovery.

By applying this path to a specific case country, it has been determined that Kazakhstan aligns with the configuration path R13. Although the government of Kazakhstan did not provide any support for debt and contract relief and did not mandate workplace closures, there has been no observed increase in incidence rates as of December 31, 2021, and the utilization rate of infectious beds remains at a low 17% (49). The high robustness of Kazakhstan’s PHERS can be attributed to stringent activity-restriction measures, such as limiting gatherings to fewer than 100 people and imposing restrictions on domestic movement (58). In addition, the government implemented a state of emergency on March 16, 2020, closing borders and restricting entry and exit between cities, limiting the free movement of residents (59).

#### 4.2.2 Configuration sufficient for PHERS resilience in rapidity

##### 4.2.2.1 Configuration path R21: ~C1* ~ C2 * ~ T1* ~ T2* ~ E1*P1*D1

Configuration path R21 is converted into the following variables: ~workplace closure (~C1) * ~ restrictions on public gatherings (~C2) * ~ restrictions on internal movement (~T1) * ~ International travel control (~T2) * ~ debt and contract relief (~E1) * face covering (P1) * testing policy (D1). This path identifies early viral testing as the core condition for achieving high rapidity in PHERS. The effectiveness of this strategy is further reinforced by widespread public adherence to personal protective measures, such as wearing masks, which effectively mitigate asymptomatic transmission in high-risk public settings and contribute to the expedited stabilization of infection rates. Among the 40 case countries analyzed, 27 have implemented open public testing programs. The majority of these nations have demonstrated recovery rates that surpass the global average, thereby highlighting the significance of these synergistic interventions.

By applying this path to specific countries, it has been determined that Bolivia and Madagascar fall under the configuration path R21. Notably, both Bolivia and Madagascar instituted mandates for the use of face coverings in all public spaces and implemented free public virus testing programs. However, these countries did not enforce stringent mandatory restrictions on the containment measures, closures, travel control nor did they provide economic support. Thus, their PHERS with rapid recovery may contributes to the early detection and isolation of cases. Empirical evidence shows that community-based interventions and public awareness campaigns have fostered the sense of public responsibility in combating the virus (60), thereby ensuring the effectiveness of accessible non-pharmaceutical interventions in some developing countries, such as Bolivia and Madagascar.

##### 4.2.2.2 Configuration path R22: C2* ~ C3* ~ T1* ~ T2* ~ E1*P1*D1

Configuration path R22 is converted into the following variables: restrictions on public gatherings (C2) * ~ stay-at-home restrictions (~C3) * ~ restrictions on internal movement (~T1) * ~ International and domestic travel (~T2) * ~ debt and contract relief (~E1) * face covering (P1) * testing policy (D1). The path emphasizes strategies on restrictions on public gatherings, face covering, and testing policy.

By applying this path to specific cases, it is found that solution R22 applies to Norway and Denmark. As determined by applying this path to specific cases, Denmark and Norway are able to successfully respond to the COVID-19 pandemic as a result of their unified leadership, reliable medical resources, and efficient public services, as well as their scientific decision-making and social responsibility in the process of policy implementation (61). In the subsequent response, these two nations have also continuously amended their measures, strengthened social distancing, promoted protective measures, and effectively contained the spread of the virus. According to the findings of this study, Norway and Denmark will place a greater emphasis on self-protection and testing.

##### 4.2.2.3 Configuration path R23: C1*C2* ~ C3* ~ T1*E1*P1*D1

Configuration path R23 is converted into the following variables: workplace closure (C1) * restrictions on public gatherings (C2) * ~ Stay-at-home restrictions (~C3) * ~ restrictions on internal movement (~T1) * debt and contract relief (E1) * face covering (P1) * testing policy (D1). This path indicates that during COVID-19, the PHERS should strengthen the blockade, improve the serviceability of the PHERS, and provide certain financial support to improve the PHERS’ rapidity. By applying this path to specific cases, it is discovered that solution R23 applies to Ireland, Hungary, and Portuguese.

Ireland, Hungary, and Portugal have all adopted stringent policies for the prevention of epidemics, including restrictions on gatherings, mandatory curfews, and the promotion of protective measures. Moreover, all three nations actively engage in public relations and education, providing early warnings, prevention and control plans, and other aspects to the public. The authorities of the three countries place a high priority on epidemic reporting and supervision, enhance the efficiency and precision of epidemic surveillance through technological means, track changes in the epidemic promptly, and take targeted actions (62). In conclusion, Ireland, Hungary, and Portugal have taken distinct and similar measures to prevent the spread of the disease. They are committed to reducing the risk of epidemics and achieving a balance between the economy and prevention through continuous adjusting and improving their programs.

## 5 Discussion

### 5.1 Theoretical contribution

The proposed theoretical framework for characterizing resilience strategies for PHERS to combat the COVID-19 crisis is comprehensive and innovative. The majority of previous studies on PHERS emphasized unilateral response strategies in response to epidemic crises, such as material emergencies, economic aid, and medical technology (63, 64). Evidence from the COVID-19 crisis indicates that promoting PHERS resilience requires a configuration of multiple strategies (37). For example, WHO/Europe proposed a framework for enhancing the health system’s resilience from the perspectives of governance, financing, resources, and service delivery (65). This framework emphasized the resilience strategies at the macro system level while omitting protection and response at the individual level. Existing research has demonstrated that vaccination and mask use are crucial to the COVID-19 pandemic response (32), and has also confirmed the necessity of integrating both institutional and grassroots strategies in public health to enhance system-wide resilience (66). Consequently, this study combines these fundamental operational resilience strategies, including containment and closures, travel control, economic support, public personal protection, early detection, and rapid response, and develops a comprehensive theoretical framework for describing resilience strategies for PHERS in response to epidemic crises. The configuration analysis of this study also validates the applicability of this theoretical framework. This framework provides public health emergency response organizations and governments with comprehensive governing principles to facilitate an orderly response to epidemic emergencies such as the COVID-19 pandemic.

This study employs the fsQCA to explore how the configuration-based influence of the selected strategies on PHERS resilience. Compared to conventional causal inference methods (i.e., linear models, and correlation analysis), QCA is ideally adapted to answering the research questions posed in this study. First, the application of fsQCA focuses on the relationship between variables, which facilitates the identification of the configuration effects between PHERS strategies. Specifically, QCA leverages various explanatory logics to verify the efficacy, transferability, and overlapping perspectives of results to illustrate the interactions between strategies and clarify core strategies (67). In addition, QCA can deal with small to medium-sized datasets and imbalanced information to produce reasonable results via multi-case comparative analysis. Combining the obtained configuration results with specific cases yields high accuracy and consistency, which also demonstrates the feasibility of using QCA in this study.

### 5.2 Practical implications

**Figure 3:**
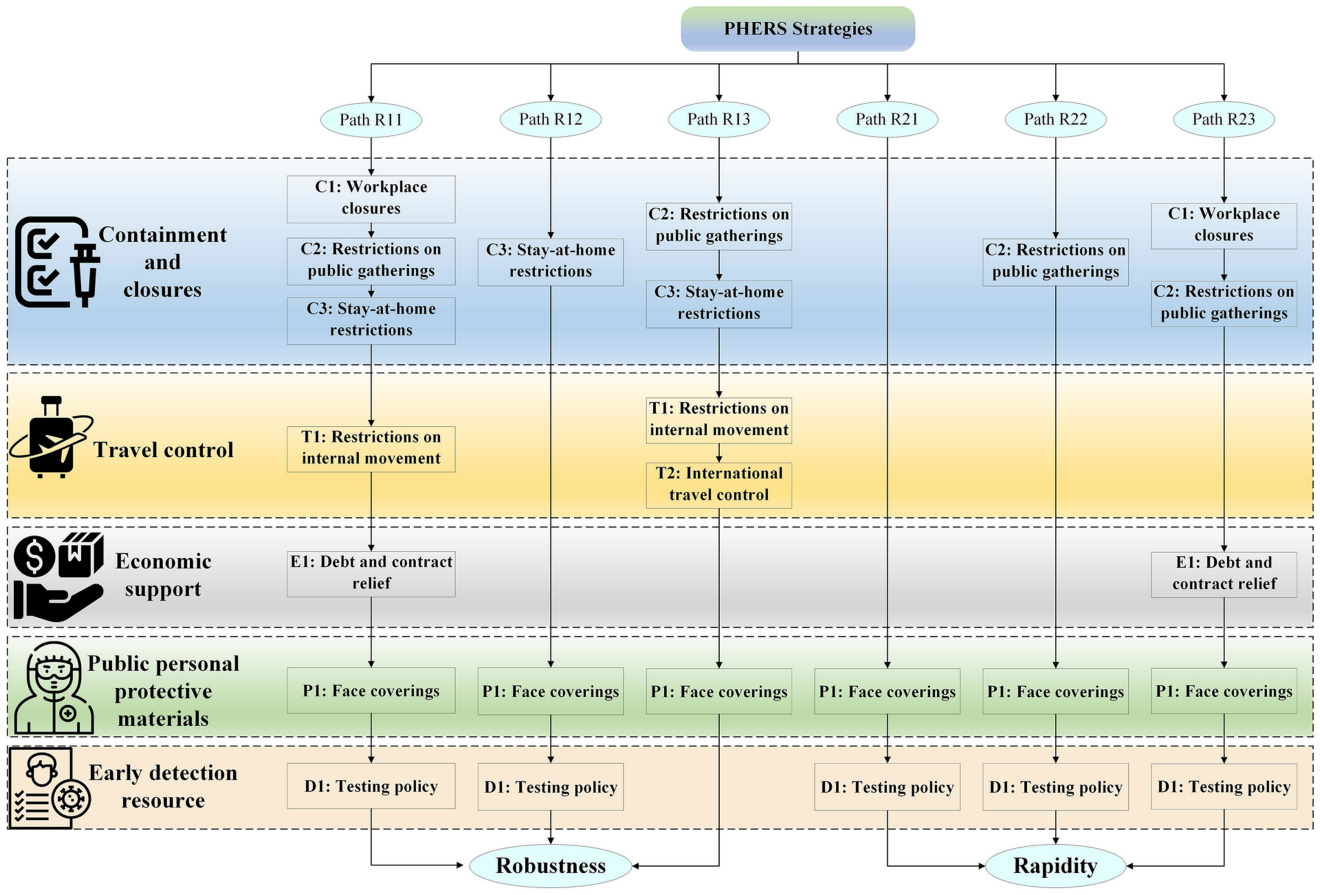
Configuration path diagram of resilience strategies for PHERS.

Due to the vast differences in medical systems, cultures, and political systems, no singular strategy can be applied universally to improve PHERS resilience in all countries. The multiple cases in this study also illustrate that the PHERS in various countries have their response strategies during the COVID-19 crisis, resulting in varying levels of resilience. Based on the explored six configuration paths (Figure 3), several practical implications for promoting PHERS resilience while accounting for country-specific differences are discussed.

For robustness, the analysis highlights stay-at-home restrictions as a shared core condition. Countries like China and Russia achieved high robustness by implementing comprehensive and stringent measures, including prohibiting international and domestic travel, which were enabled by their political systems (68). In contrast, countries with limited healthcare resources, such as Madagascar and Kazakhstan (56), prioritized isolation and low-cost containment measures, often relying on traditional medicine and local enforcement. These examples suggest that countries with large populations may require stricter travel controls, while smaller countries might achieve similar robustness through targeted, resource-efficient strategies.

For rapidity, the critical components across all configurations include face covering and positive testing. Countries with under-resourced health systems (e.g., India) often adopt basic preventive approaches, such as promoting herbal medicines, increasing medical supplies (69), enhancing isolation (70) and blocking to combat the spread of COVID-19. Meanwhile, countries with advanced infrastructures (e.g., Switzerland) leverage widespread testing and digital surveillance to respond quickly and efficiently (71). These cases emphasize that rapid recovery depends not solely on the intensity of restrictions, but on the timely deployment of appropriate interventions.

Importantly, the results indicate that PHERS resilience depends not only on the severity of policies but more critically on how well interventions are strategically configured and contextually adapted. For example, although Brazil implemented relatively strict containment measures, it failed to achieve a high level of PHERS resilience (72), which highlights the risks of mismatches between strategy and system capacity as well as the limitations of overly rigid approaches. Rather than pursuing maximal stringency, policymakers should prioritize flexible, evidence-based strategies tailored to specific national conditions. Accordingly, this study proposes several broadly applicable recommendations: restrict non-essential mobility through transparent and enforceable legal frameworks; improve early detection through widespread testing and contact tracing; and provide financial and logistical support to reduce the burden on affected populations. These integrated strategies not only improve the effectiveness of emergency responses but also strengthen institutional credibility and public trust during health crises.

## 6 Conclusion

Using the fsQCA approach, this study reveals the configurational strategies affecting PHERS resilience and then presents several distinct findings in the context of Covid-19 crisis. Unlike prior research that primarily focused on individual strategies or country-specific cases, this study adopts a cross-national, configuration-oriented perspective to uncover how combinations of policy measures contribute to high levels of robustness and rapidity. The study proposes a comprehensive theoretical framework grounded in the 4R resilience theory and analyzes PHERS data from 40 countries using robustness and rapidity as outcome indicators. The findings identify six effective configuration paths: three promoting robustness and three enhancing rapidity, which collectively demonstrate that limiting people’s movement and aggregation, as well as early detection and rapid response, are crucial for PHERS to promote high resilience. Importantly, such restrictive measures should be implemented in accordance with ethical principles, ensuring that public health interventions are proportionate, equitable, and procedurally fair. This study complements existing literature by offering an integrated view of resilience strategies and validating their combined effectiveness through empirical data. It provides actionable guidance for policymakers: rather than maximizing the intensity of a single policy, governments should focus on context-adapted combinations of interventions tailored to their national systems. Notably, resilience strategies must be adapted to each country’s specific conditions, as medical systems, cultures, and political structures vary significantly. The study’s novelty lies in its methodological contribution, demonstrating how fsQCA can capture the causal complexity of health policy performance across countries. It also has practical implications for enhancing the preparedness and responsiveness of PHERS under future pandemic threats.

Future research may extend this work by incorporating longitudinal data to observe how configuration effectiveness evolves over time, or by integrating machine learning and causal inference models to further explain why specific configurations succeed. Moreover, resilience strategies at the community or regional level should be explored to develop a more localized understanding of PHERS performance.

## Data Availability

The original contributions presented in the study are included in the article/Supplementary material, further inquiries can be directed to the corresponding authors.
