# Supplementary material for: Toward resilience for public health emergency response system during COVID-19: qualitative comparative analyses of 40 countries
Source: Front Public Health. 2025 Sep 18;13:1652309. doi: 10.3389/fpubh.2025.1652309 (PMC12488601; doi:10.3389/fpubh.2025.1652309)
Supplement: Supplementary file 1 [file Table_1.DOCX]

Appendix A

**Table S1: All variables data from 40 countries**

| ID | Country | Condition variables | | | | | | | | | Outcome variables | | | |
| --- | --- | --- | --- | --- | --- | --- | --- | --- | --- | --- | --- | --- | --- | --- |
|  |  | Containment and closures | | | | Travel control | | Economic support | Public personal protective materials | Early detection resource | PHERS resilience | | | |
|  |  | C1 Workplace closures | C2 Restrictions on public gatherings | C3  Stay-at-home restrictions | T1 Restriction on internal movement | | T2 International and domestic travel | E1 | P1 | D1 Testing policy | Robustness value | R1 | Rapidity value | R2 |
|  |  |  |  |  |  |  |  | Income support | Face coverings |  |  |  |  |  |
| 1 | China | 1 | 1 | 1 | 1 | | 0.33 | 1 | 0.67 | 1 | 99.99% | 1 | 4.667 | 1 |
| 2 | Japan | 0.33 | 0.33 | 0.33 | 0.5 | | 1 | 1 | 0.33 | 1 | 98.62% | 1 | 21.333 | 1 |
| 3 | South Korea | 0.67 | 1 | 0 | 0 | | 0.67 | 0.5 | 0.67 | 1 | 98.77% | 1 | 5.333 | 1 |
| 4 | Thailand | 0.67 | 1 | 0 | 0.5 | | 0.67 | 1 | 0.67 | 1 | 96.83% | 1 | 50.667 | 0 |
| 5 | Kazakhstan | 0.33 | 1 | 0.67 | 1 | | 0.67 | 0 | 0.67 | 0.33 | 94.40% | 1 | 39 | 0 |
| 6 | Pakistan | 0.67 | 0.67 | 0.33 | 1 | | 0.67 | 1 | 0.67 | 1 | 99.36% | 1 | 50.333 | 0 |
| 7 | India | 0 | 1 | 0.67 | 1 | | 0.33 | 0.5 | 0.67 | 0.67 | 97.52% | 1 | 94.667 | 0 |
| 8 | the US | 0.67 | 1 | 0.33 | 0.5 | | 0.67 | 0 | 0.67 | 1 | 83.98% | 0 | 71.667 | 0 |
| 9 | Mexico | 0.33 | 0 | 0.33 | 1 | | 0.33 | 0.5 | 0.67 | 0.33 | 96.95% | 1 | 93 | 0 |
| 10 | Argentina | 0.33 | 0.33 | 0.33 | 0 | | 0.33 | 0.5 | 1 | 0.67 | 87.49% | 0 | 55.667 | 0 |
| 11 | Peru | 0.67 | 1 | 0.67 | 0.5 | | 0.67 | 0.5 | 1 | 1 | 93.19% | 1 | 52.333 | 0 |
| 12 | Uruguay | 0.33 | 0.33 | 0 | 0 | | 0.33 | 0.5 | 0.67 | 1 | 88.25% | 0 | 20.667 | 1 |
| 13 | Bolivia | 0.33 | 0 | 0 | 0 | | 0.33 | 0 | 1 | 1 | 95.02% | 1 | 32.667 | 1 |
| 14 | Colombia | 0.67 | 1 | 0.33 | 0 | | 1 | 0.5 | 1 | 0.67 | 90.01% | 0 | 49 | 0 |
| 15 | Brazil | 0.67 | 1 | 0.33 | 1 | | 0.67 | 0.5 | 0.67 | 1 | 89.64% | 0 | 88.667 | 0 |
| 16 | Canada | 1 | 1 | 0.33 | 1 | | 1 | 0.5 | 0.67 | 0.33 | 94.51% | 1 | 28.667 | 1 |
| 17 | Russia | 0.67 | 0.67 | 0.67 | 1 | | 0.67 | 1 | 0.67 | 1 | 92.80% | 1 | 109.67 | 0 |
| 18 | Turkey | 0.67 | 0 | 0 | 1 | | 0.33 | 0 | 1 | 0.33 | 88.94% | 0 | 43 | 0 |
| 19 | Australia | 0 | 1 | 0 | 1 | | 0.67 | 0 | 0.67 | 0.67 | 98.61% | 1 | 7.333 | 1 |
| 20 | Norway | 0.67 | 1 | 0.33 | 0 | | 0.33 | 0 | 0.67 | 1 | 92.90% | 1 | 8.333 | 1 |
| 21 | the UK | 0.67 | 1 | 0 | 0 | | 0.33 | 0.5 | 0.67 | 0.67 | 80.35% | 0 | 85.333 | 0 |
| 22 | Germany | 1 | 1 | 0.33 | 1 | | 0.67 | 0 | 0.67 | 1 | 91.48% | 1 | 43.333 | 0 |
| 23 | France | 0.67 | 1 | 0 | 1 | | 0.67 | 0 | 0.67 | 1 | 85.53% | 0 | 46 | 0 |
| 24 | Ireland | 0.67 | 1 | 0 | 0 | | 0.67 | 1 | 0.67 | 0.67 | 85.76% | 0 | 32.667 | 1 |
| 25 | Netherlands | 0.67 | 1 | 0.67 | 0 | | 0.67 | 0 | 0.67 | 1 | 81.89% | 0 | 33.667 | 1 |
| 26 | Romania | 0.33 | 0.67 | 0.67 | 0.5 | | 0.67 | 0.5 | 0.67 | 1 | 90.49% | 1 | 51.333 | 0 |
| 27 | Belgium | 0.67 | 1 | 0 | 0 | | 0.67 | 0.5 | 0.67 | 1 | 81.87% | 0 | 21 | 1 |
| 28 | Hungary | 0.67 | 0.67 | 0 | 0 | | 0.33 | 1 | 0.67 | 1 | 86.93% | 0 | 32 | 1 |
| 29 | Finland | 0.33 | 1 | 0 | 0 | | 0.33 | 0.5 | 0 | 0.67 | 95.12% | 1 | 9.333 | 1 |
| 30 | Sweden | 0.33 | 1 | 0 | 0 | | 0.67 | 0.5 | 0 | 0.67 | 87.12% | 0 | 22.667 | 1 |
| 31 | Switzerland | 0.67 | 1 | 0 | 0 | | 0.67 | 0 | 0.67 | 1 | 84.76% | 0 | 56.667 | 0 |
| 32 | Austria | 0.67 | 1 | 0.67 | 0 | | 0.67 | 1 | 0.67 | 1 | 85.97% | 0 | 160.67 | 0 |
| 33 | Poland | 0.67 | 0 | 0 | 0 | | 0.67 | 1 | 0.67 | 0.67 | 89.12% | 0 | 36.667 | 0 |
| 34 | Spain | 1 | 1 | 0 | 1 | | 0.67 | 1 | 1 | 1 | 86.09% | 0 | 34.333 | 1 |
| 35 | Portuguese | 0.67 | 1 | 0 | 0 | | 0.33 | 1 | 0.67 | 1 | 86.61% | 0 | 33 | 1 |
| 36 | Italy | 1 | 0.33 | 0.67 | 1 | | 0.67 | 1 | 0.67 | 1 | 90.08% | 0 | 33.333 | 1 |
| 37 | Denmark | 0.67 | 0.67 | 0 | 0 | | 0.33 | 0 | 0.67 | 1 | 86.15% | 0 | 13.333 | 1 |
| 38 | South Africa | 0.67 | 0.33 | 0 | 0 | | 0.33 | 0.5 | 0.67 | 1 | 94.31% | 1 | 85.333 | 0 |
| 39 | Somalia | 0.33 | 1 | 0.33 | 0 | | 0.33 | 0 | 0.67 | 1 | 99.86% | 1 | 24.667 | 1 |
| 40 | Madagascar | 0 | 0 | 0.67 | 0 | | 0.33 | 0 | 1 | 1 | 99.83% | 1 | 21 | 1 |
